# Endothelial and smooth muscle cells derived from human cardiac explants demonstrate angiogenic potential and suitable for design of cell-containing vascular grafts

**DOI:** 10.1186/s12967-017-1156-1

**Published:** 2017-03-03

**Authors:** I. S. Zakharova, M. K. Zhiven’, Sh. B. Saaya, A. I. Shevchenko, A. M. Smirnova, A. Strunov, A. A. Karpenko, E. A. Pokushalov, L. N. Ivanova, P. I. Makarevich, Y. V. Parfyonova, E. Aboian, S. M. Zakian

**Affiliations:** 1 The Federal Research Center Institute of Cytology And Genetics, The Siberian Branch of the Russian Academy of Sciences, Novosibirsk, Russian Federation; 2 Institute of Chemical Biology and Fundamental Medicine, The Siberian Branch of the Russian Academy of Sciences, Novosibirsk, Russian Federation; 3 Siberian Federal Biomedical Research Center, Ministry of Health Care of Russian Federation, Novosibirsk, Russian Federation; 4 Novosibirsk State University, Novosibirsk, Russian Federation; 5 Laboratory of Angiogenesis, Russian Cardiology Research and Production Complex, Moscow, Russian Federation; 6 Laboratory of gene and cell therapy, Institute of regenerative medicine, Lomonosov Moscow State University, Moscow, Russian Federation; 7 Faculty of Medicine, Lomonosov Moscow State University, Moscow, Russian Federation; 8 Division of Vascular Surgery, Palo Alto Medical Foundation, Burlingame, USA

**Keywords:** Endothelial cells, Smooth muscle cells, Human cardiac explant, Polycaprolactone, Chitosan, Tissue-engineered vascular grafts

## Abstract

**Background:**

Endothelial and smooth muscle cells are considered promising resources for regenerative medicine and cell replacement therapy. It has been shown that both types of cells are heterogeneous depending on the type of vessels and organs in which they are located. Therefore, isolation of endothelial and smooth muscle cells from tissues relevant to the area of research is necessary for the adequate study of specific pathologies. However, sources of specialized human endothelial and smooth muscle cells are limited, and the search for new sources is still relevant. The main goal of our study is to demonstrate that functional endothelial and smooth muscle cells can be obtained from an available source—post-surgically discarded cardiac tissue from the right atrial appendage and right ventricular myocardium.

**Methods:**

Heterogeneous primary cell cultures were enzymatically isolated from cardiac explants and then grown in specific endothelial and smooth muscle growth media on collagen IV-coated surfaces. The population of endothelial cells was further enriched by immunomagnetic sorting for CD31, and the culture thus obtained was characterized by immunocytochemistry, ultrastructural analysis and in vitro functional tests. The angiogenic potency of the cells was examined by injecting them, along with Matrigel, into immunodeficient mice. Cells were also seeded on characterized polycaprolactone/chitosan membranes with subsequent analysis of cell proliferation and function.

**Results:**

Endothelial cells isolated from cardiac explants expressed CD31, VE-cadherin and VEGFR2 and showed typical properties, namely, cytoplasmic Weibel-Palade bodies, metabolism of acetylated low-density lipoproteins, formation of capillary-like structures in Matrigel, and production of extracellular matrix and angiogenic cytokines. Isolated smooth muscle cells expressed extracellular matrix components as well as α-actin and myosin heavy chain. Vascular cells derived from cardiac explants demonstrated the ability to stimulate angiogenesis in vivo. Endothelial cells proliferated most effectively on membranes made of polycaprolactone and chitosan blended in a 25:75 ratio, neutralized by a mixture of alkaline and ethanol. Endothelial and smooth muscle cells retained their functional properties when seeded on the blended membranes.

**Conclusions:**

We established endothelial and smooth muscle cell cultures from human right atrial appendage and right ventricle post-operative explants. The isolated cells revealed angiogenic potential and may be a promising source of patient-specific cells for regenerative medicine.

**Electronic supplementary material:**

The online version of this article (doi:10.1186/s12967-017-1156-1) contains supplementary material, which is available to authorized users.

## Background

Endothelial cells (EC) and smooth muscle cells (SMC) are two major cell types of blood vessels. EC and SMC are necessary for normal circulation and involved in different physiological processes, including angiogenesis, blood pressure control, coagulation, and inflammation [1–4]. The study of EC and SMC is important for understanding the aetiology and treatment of cardiovascular and other diseases, including cancer and diabetes, since endothelial dysfunction is the basis of a broad range of pathologies [5]. Both EC and SMC are considered promising resources for regenerative medicine and cell replacement therapy [6–21].

Different cultures of endothelial and smooth muscle cells are used as models for studies of diseases. In vivo EC have high functional and structural heterogeneity depending on vessel type (artery, vein or lymphatic) and tissue [3, 14, 22–25]. Endothelial heterogeneity manifests in embryonic development, and a cytokine-dependent signalling axis, along with the influence of the extracellular matrix and mechanical forces, drives this process further in the postnatal period. Indeed, it has been shown that EC and SMC isolated from various organs express defined patterns of transcription factors, angiogenic cytokines, adhesion molecules and chemokines [3, 14, 22, 23, 26–28] that are essential to meet metabolic needs and adapt the cell to the organ environment. Therefore, isolation of EC and SMC from tissues relevant to the area of research is necessary for adequate study of specific pathologies [29]. In addition, endothelial cells and their precursors are regarded as a promising source for regenerative medicine to treat pathological conditions such as cardiac infarction, muscle ischaemia, and bone fractures [13]. Generation of sufficient vascular networks is also crucial for wound healing and successful tissue engineering.

It has been shown that the injection of EC and SMC in ischaemic organs leads to the formation of new blood vessels and promotes the recovery of the vascular network. The regenerative potential of endothelial cells is enhanced by smooth muscle cells [30, 31]. Revascularization after injection of EC and SMC may be of a paracrine nature due to release of specific angiocrine factors that stimulate angiogenesis [9, 15, 25].

Many researchers are currently seeking clinically relevant cell sources for the isolation of postnatal endothelial cells, endothelial progenitor cells [6, 13, 29, 32–36] and smooth muscle cells [7, 16, 17, 19–21]. However, sources of specialized human EC and SMC are limited, and the search for new sources is still relevant.

More than 20 years ago, two groups of authors developed methods for isolating human endothelial cells from the heart of the recipient during cardiac transplantation to study rejection of the donor organ [37, 38]. However, this source is limited and may not be useful for regenerative purposes.

The aim of this study is to evaluate the use of primary human material rich in both EC and SMC, namely, right atrial appendage and ventricular cardiac tissues discarded during surgery. The atrial appendage is traditionally used as a source of c-kit-positive cardiac stem cells [39, 40]. Additionally, in cultures derived from the atrial appendage, the presence of EC and SMC markers has been shown previously [41]. The main goal of our study is to show that functional endothelial and smooth muscle cells can be obtained from an available source—surgical discarded cardiac tissue from right atrial appendage and right ventricular myocardium. We established a method for isolation, culture and enrichment of EC and SMC from cardiac tissues. This method yields a significant number of cells (up to 10^6^) when they are cultured for 14 days in specific growth media for endothelial and smooth muscle cells. This amount is sufficient for magnetic sorting of CD31-positive endothelial cells. Endothelial and smooth muscle cells derived from human right atrial appendage and right ventricular myocardium may be a promising source of patient-specific cells for regenerative medicine purposes, especially for patients who face the prospect of surgical reoperation. For example, one application of endothelial cells is the development of cell-seeded vascular grafts, especially those under 6 mm in diameter [8, 42]. As an example of what cells isolated by the proposed method may be used for tissue engineering, we seeded the cells on a biodegradable scaffold—a polycaprolactone/chitosan (PCL/CH) blended membrane.

We believe that the right atrial appendage and right ventricular myocardium can be used as a promising patient-specific source of EC and SMC. We think that our findings will be useful to scientists working towards improving techniques for therapeutic angiogenesis and tissue regeneration.

## Methods

### Ethical statements

All procedures with human material were approved by the ethical board of the State Research Institute of Circulatory Pathology, Ministry of Healthcare of the Russian Federation (permit No. 45 by 26.12. 2014), and all donors signed an informed consent form. The animal tests were conducted in an SPF vivarium and were approved by the Institute of Cytology and Genetics of the Siberian Branch of the Russian Academy of Sciences (IC&G SD RAS) ethical board (permit No. 22.4 by 30.05.2014).

### Cell isolation and culture

Cells were isolated from post-surgically discarded samples of human right atrial appendage and right ventricle myocardium as follows: the tissue was mechanically ground into pieces (1–3 mm^3^) and then enzymatically treated in 0.1% collagenase NB solution (Life Technologies, USA) at 37 °C for 30 min. After that, the resulting mixture of cells and debris was centrifuged at 300*g* for 5 min and seeded in plastic dishes coated with human collagen IV (Sigma, USA) in culture media specific for EC or SMC–EGM-2 (Endothelial Cell Growth Medium-2) or SmGM-2 (Smooth Muscle Growth Medium-2) (both—Lonza, Switzerland). Cell culture was maintained in 5% CO_2_ at 37 °C with 1:2–1:3 passaging using TrypLE Express enzyme (Life Technologies, Denmark). The medium was replaced completely every other day; half of the culture medium was replaced daily. When primary cell culture in EGM-2 reached a monolayer, 10^6^ cells were sorted using magnetic MicroBeads (130-091-935, Miltenyi Biotec, Germany) conjugated with antibodies against human CD31. The procedure of magnetic-activated cell sorting (MACS) was conducted according to the manufacturer’s instructions.

### Preparation of chitosan/PCL polymer membranes

Chitosan/PCL membranes for cell seeding were prepared as described earlier [43–45] with certain modifications. Stock solutions were made: 1) 1 wt% chitosan with 85% deacetylation degree (Sigma–Aldrich, USA) in 0.5 M acetic acid, 2) 10 wt% PCL in glacial acetic acid, and 3) the additional dilutions of PCL listed below, also prepared in glacial acetic acid. Resulting PCL stock solutions were mixed with 3 ml of 1% chitosan to obtain the following combinations in a total volume of 10 ml:

PCL25 (PCL:chitosan 1:3) = 10 ml 0.1% PCL + 3 ml 1% chitosan

PCL50 (PCL:chitosan 1:1) = 10 ml 0.3% PCL + 3 ml 1% chitosan

PCL75 (PCL:chitosan 3:1) = 10 ml 0.9% PCL + 3 ml 1% chitosan

The obtained solutions (PCL25, PCL50, PCL75) were poured onto culture dishes (2 ml/10 cm^2^) and allowed to air-dry in a thermostat (55 °C) for 24 h until film formation. Membranes on dishes were neutralized for 30 min either with alkaline (0.5 M NaOH [2% w/v] for 30 min) or with alkaline/ethanol mixture (0.5 M NaOH in 80% ethanol followed by 3 washes in 80% ethanol). Finally, dishes with membranes were washed in PBS (phosphate-buffered saline), UV-sterilized for 40 min and placed in a CO_2_ incubator with 5% CO_2_ at 37 °C.

### Immunofluorescent staining of cells

Passage 1 EC and SMC were grown to confluence on plastic dishes or on chitosan/PCL membranes. After that, they were fixed with 4% PFA (paraformaldehyde) for 10 min, permeabilized with 0.05% Triton X-100 for 10 min, and blocked with 1% BSA (bovine serum albumin). The cells were stained with primary antibodies overnight at 4 °C, washed with PBS and incubated with secondary antibodies for 1 h at room temperature. The stained cells were analysed with an inverted fluorescence microscope (Nikon Ti-E) using Nikon AR software.

The following primary antibodies were used: anti-human CD31 (M0823, DAKO, 1:50), anti-α-SMA (DAKO, M0851, 1:50), anti-smooth muscle myosin heavy chain 11 (Abcam, ab82541, 1:500), anti-human CD90 (eBioscience, 14090982, 1:100), anti-Von Willebrand factor (Abcam, ab6994, 1:200), anti-fibronectin (Abcam, ab6328, 1:200), anti-elastin (Abcam, ab21610, 1:200), and anti-collagen IV (Life Span, 1:200).

The following secondary antibodies were used: Alexa Fluor 568 goat anti-mouse IgG1 (Life Technologies, A21124, 1:400), Alexa Fluor 488 goat anti-mouse IgG1 (Life Technologies, A21121, 1:400), Alexa Fluor 568 goat anti-mouse IgG2a (Life Technologies, A21134, 1:400), Alexa Fluor 568 goat anti-mouse IgG (H + L) (Life Technologies, A11031, 1:400), and Alexa Fluor 488 goat anti-mouse IgG (H + L) (Life Technologies, A11029, 1:400).

### Flow cytometry quantitative analysis of cell populations

Cells in EGM-2 were used for fluorescence-activated cell sorting (FACS) analysis at the second passage before MACS and at the third passage after separation of CD31+ cells by MACS. Cells in SmGM-2 were used for FACS analysis at the second and fifth passages. The viability of cells was assessed by Trypan Blue (T10282, Invitrogen, USA) using a Countess Automated Cell Counter (C10227, Korea) and was more than 95%. The cells were detached with enzyme-free cell dissociation buffer (13151014, Gibco). Non-fixed cells were used for direct staining by fluorochrome-conjugated antibodies: anti-human CD31-APC (17-0319-42, eBioscience), anti-human VEGFR2-PE (560494, BD Biosciences), and anti-human CD90 (17-0909-42, eBioscience). Isotype-matched antibodies served as negative control. For indirect staining with anti-α-SMA antibodies (DAKO, M0851), cells were fixed with 4% PFA for 10 min, permeabilized with 0.1% TWEEN for 20 min, blocked with 1% BSA and incubated with primary antibodies overnight at 4 °C. Then, the cells were washed with PBS and incubated with Alexa Fluor 488 goat anti-mouse IgG (H + L) (Life Technologies, A11029) secondary antibody for 1 h at room temperature. The secondary antibody without the primary antibody was used as negative control. Cell populations were analysed on a FACS Canto II device using FACS Diva software. Assays were run in triplicate, and the data are presented as the mean ± standard deviation. Antibody dilutions and cell preparations were made according to the manufacturer’s instructions.

### Analysis of endothelial cell ultrastructure

Endothelial cells from passage 2 were cultured on specific plastic films (Agar Scientific, Essex, UK) for 2, 4 and 8 days after MACS separation. EC attached to the films were fixed with 2.5% glutaraldehyde in cell culture medium for 15 min at room temperature and removed to a fresh solution of 2.5% glutaraldehyde in 0.1 M sodium cacodylate (pH 7.4) for 1 h incubation at room temperature with light agitation. Cells were washed three times for 5 min each in 0.1 M sodium cacodylate and post-fixed for 1 h in a 1% aqueous solution of osmium tetroxide containing 0.8% (w/v) of potassium ferricyanide (K_3_[Fe(CN)_6_]). After being washed with deionized water, the samples were incubated overnight at 4 °C in a 1% aqueous solution of uranyl acetate. The next day, the cells were washed once with deionized water, dehydrated in an ethanol series (30%, 50%—5 min, 70%, 96%—10 min, 100%—15 min) and acetone (twice, 20 min), and embedded in Agar 100 Resin (Agar Scientific, Essex, UK). Final polymerization of samples was induced by a 3-day incubation at 60 °C. Semi-thin sections were obtained with a Reichert-Jung Ultracut microtome, stained with methylene blue and analysed with a Zeiss Axioskop 40 light microscope. Ultra-thin sections were made using a Leica Ultracut microtome and stained with Reynolds lead citrate. Sections were examined with a JEOL JEM-100SX transmission electron microscope at the Interinstitutional Center for Microscopic Analysis of Biological Objects (IC&G SD RAS, Novosibirsk, Russia).

### Cytokine pattern analysis

A total of 10^6^ sorted and unsorted EC were grown in EGM-2 for 24 h on 10 cm dishes. After that, the medium was removed, the cells were washed with PBS three times, and unsupplemented basal EBM was added for 120 h to obtain conditioned medium. Cytokine pattern (vascular endothelial growth factor, VEGF; stromal cell-derived factor 1α, SDF1α; hepatocyte growth factor, HGF; epidermal growth factor, EGF; fibroblast growth factor 2, FGF2) was analysed by enzyme-linked immunosorbent assay (ELISA) using Quantikine kits (R&D Systems, USA).

### Functional characteristics of EC and SMC in a Matrigel plug model

An in vivo Matrigel angiogenesis assay was conducted as previously described [25, 46]. Female immunodeficient (severe combined immunodeficiency, SCID) mice (6–8 weeks, 22–28 g) were used for experiments. Animals were kept in the SPF vivarium of the IC&G SD RAS (Novosibirsk, Russia). EC and SMC were used at passage 5–7, and 5 × 10^5^ cells in 50 µl PBS were mixed with 50 µl of Matrigel (BD Biosciences, USA) immediately before injection. A total of 100 µl of suspension was injected subcutaneously into the abdominal area of the animal. The animals (n = 15) were divided into three groups: a control group receiving only PBS (n = 5), a Matrigel + EC group (n = 5) and a group that received a Matrigel + EC/SMC 1:1 mixture injection (n = 5). The endothelial cells were labelled with vital MitoTracker Deep Red FM (M22426, Molecular Probes, USA). The animals were sacrificed after 14 days. The Matrigel plug was isolated and frozen in TissueTek (Sakura, USA), and 10 µm sections of the vascularized implant were obtained on a Leica HM550 cryotome (Leica, Germany). Prepared sections were stained with fluorescently tagged isolectin B4 to visualize the vasculature, and isolectin-positive structures were captured in 10 fields of view (FOVs) on a Nikon Ti-E microscope. Structure counts were obtained using Angio Tool software [47], and the results were analysed using a Wilcoxon test with the Bonferroni correction in R version 3.3.1 (R Core Team, Austria) [48]. Representative photos were performed using confocal microscope LSM 780 NLO (Zeiss) at the Microscopy Center of the Institute of Cytology and Genetics, SB RAS, Russia.

### Fluorescent in situ hybridization (FISH)

#### Pretreatment of cryosections before FISH

Slides with cryosections were incubated in xylene for 10 min followed by rehydration in 100% ethanol 2 times for 5 min and 90, 80, 70–75, 50% ethanol for 5 min each. Then, the slides were washed in PBS for 15 min and incubated in 1% Triton X-100 overnight. The next day, the slides were washed in PBS for 15 min, transferred into a Coplin jar with prewarmed (80 °C) 10 mM sodium citrate buffer in a water bath, and incubate for 1 h. The slides were washed by soaking into a Coplin jar with PBS followed by incubation in prewarmed (37 °C) 0.01% pepsin for 10 min. Then, the slides were transferred into a Coplin jar with 1% BSA and washed in 2X SSC (saline-sodium citrate) 2 times for 5 min followed by incubation in Triton X-100 (0.7%)/HCl (0.1 N). Finally, the slides were washed in 2X SSC 2 times for 5 min.

#### Probe preparation

Human Cot-1 DNA (15279011, Thermo Fisher Scientific) and mouse Cot-1 DNA (18440016, Thermo Fisher Scientific) were labelled with biotin 16-dUTP (11093070910, Roche) or digoxigenin-11-dUTP (11558706910, Roche) in 30 PCR cycles by DOP-PCR (degenerate oligonucleotide-primed PCR) with MW6 primer [49]. Human and mouse Cot-1 DNA and labelled probes (100 ng of each) were mixed with 1 mg/ml salmon sperm DNA, followed by addition of 2 volumes of 100% ethanol and complete desiccation in a speed vac. The desiccated probes were dissolved in 10 µl of hybridization solution containing 50% formamide, 10% dextran sulphate, 2X SSC (1X SSC: 0.15 M NaCl, 0.015 M sodium citrate, pH 7.0), and 1% TWEEN 20 for 1 h at 37 °C in a water bath. The probes were denatured in the hybridization solution for 5 min at 75 °C and transferred to ice quickly.

#### Slide preparation, in situ hybridization and washes

The slide-mounted cryosections were denatured in prewarmed 70% formamide/2X SSC for 20 min at 70 °C followed by incubation in 2X SSC for 2 min. The slides were dried at room temperature (r.t.). Ten microliters of probe was pipetted onto a prewarmed coverslip and picked up with a slide followed by incubation in a humid chamber in a water bath at 37 °C overnight. The next day, the slides were washed in 2X SSC/50% formamide 4 × 3 min at 37 °C in a water bath, then in 2X SSC 4 × 3 min at 37 °C and transferred to 4X SSC/0.1% TWEEN 20 at r.t.

#### Detection

Biotinylated probes were detected with a fluorescein-avidin (A-2011, Vector Laboratories)/biotinylated anti-avidin system (BA-0300, Vector Laboratories) and digoxigenin-labelled DNA was visualized with anti-digoxigenin rhodamine Fab fragments (11207750910, Roche). The nuclei were counterstained with 40,6-diamino-2-phenylindol in antifade (Vectashield, H-1200, Vector Laboratories) and then visualized using a Nikon Ni-E microscope.

### XTT proliferation assay

Endothelial cells were seeded at a density of 2.5*10^4^/cm^2^ in EGM-2 on plastic or chitosan/PCL membranes. Endothelial cell proliferation on chitosan/PCL was estimated by an XTT test (2,3-bis-(2-methoxy-4-nitro-5-sulfophenyl)-2H-tetrazolium-5-carboxanilide) (11465015001, Roche, USA) according to the manufacturer’s instructions at 22 h after the reagents were added. The results were estimated using a scanning spectrophotometer 2030 Multilabel Reader Victor X3 (Perkin-Elmer, USA) at 490 nm wavelength.

### Evaluation of EC angiogenic potential in 3D Matrigel tubulogenesis assay

CD31+ (positive) sorted EC were stained with the vital mitochondrial dye TMRM (tetramethylrhodamine, methyl ester) (Life Technologies, USA) and cultured until 40–60% confluence. After that, the growth medium was aspirated and a 1:1 mixture of Matrigel (BD Biosciences, USA) with growth medium was prepared on ice. The obtained mixture was poured on top of the cells, and after 24 h of incubation, the formation of capillary-like structures was observed. Pictures were obtained on an inverted fluorescence microscope (Nikon Ti-E) by the Z-stack method, applying the Advanced Research software 6D module.

### Cell seeding and viability assay

The proliferation of EC on different surface types was estimated by cell counts obtained at 1, 8 and 12 days after seeding on different matrices. The cells were labelled with TMRM vital mitochondrial dye (Life Technologies, USA) or NucBlue vital nuclear dye (Life Technologies, USA). Cells were counted in 5 random FOVs with a 10× objective using an inverted fluorescence microscope (Nikon Ti-E, Nikon, Japan) and NIS Advanced Research software (Nikon, Japan). All experiments were conducted in triplicate for statistical analysis.

### Preparation of sections from membranes seeded with endothelial cells

For histological study, we chose CD31+ EC cultured on PCL75 membranes, which were fixed with 4% PFA as described above. The membrane with fixed cells was separated from the dish with tweezers and frozen in TissueTek; after that, 10 µm sections were prepared on a Cryostat Microm HM 550 (Microm International GmbH, Germany) in the Multiple-access Center for Microscopy of Biological Subjects (Institute of Cytology and Genetics, Novosibirsk, Russia).

### Statistical analysis

RStudio version 3.3.1 [48] and Microsoft Excel version 14.0.6023.1000 were used for data analysis. Data are presented as the mean ± SD. The Wilcoxon test, with Bonferroni correction where required, was used for comparison between the two groups. p < 0.05 was considered significant.

## Results

### Specific enrichment of primary culture from cardiac explants for EC and SMC

Human cardiac explants consist of many cell types. The goal of our study was to establish separate endothelial and smooth muscle cell cultures from right atrial appendage and right ventricle myocardium explants. Thus, enzymatically derived cells and the remaining explant tissue were divided into two equivalent parts. One part was placed into the specific medium for the growth of endothelial cells (EGM-2), and the other into the medium for the growth of smooth muscle cells (SmGM-2).

**Fig. 1:**
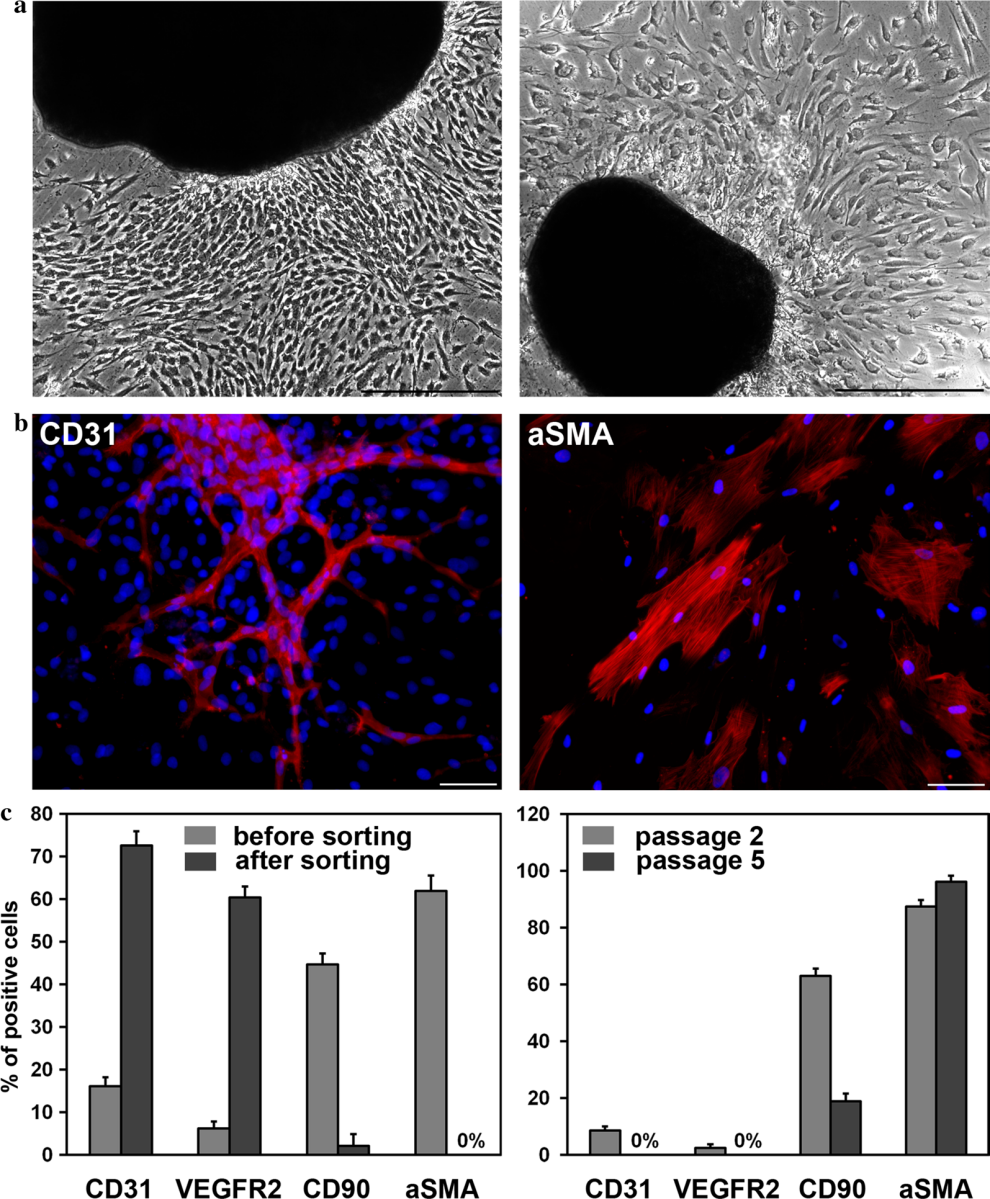
Cells isolated from cardiac explants in culture. **a** Morphology of primary cells migrated from cardiac explants. Phase contrast images of cells cultivated in an endothelial growth medium (*left panel*) and cells derived in a smooth muscle growth medium (*right panel*). *Scale bar* 100 μm. **b** CD31-positive endothelial (*left panel*) and αSMA-positive smooth muscle (*right panel*) cells were detected by immunofluorescent staining of primary cardiac explant cultures growing in endothelial or smooth muscle medium, respectively. *Scale bar* 100 μm. **c** Flow cytometric analysis of surface markers. Comparison of cells cultivated in endothelial growth medium before and after MACS separation (*left panel*). Comparison of cells cultivated in smooth muscle cell growth medium at the second and fifth passages (*right panel*)

Primary culture cell morphology is shown in Fig. 1a, and further analysis by immunostaining detected expression of CD31 and α-smooth muscle actin (αSMA) in EC and SMC, respectively, cultured in specific media (Fig. 1b). Flow cytometry results showed that, prior to sorting, the proportion of CD31-positive endotheliocytes in primary culture grown on EGM-2 comprised 16.1 ± 2.1% (Fig. 1c, left panel), and 44.7 ± 2.5% of those cells were detected to be CD90 positive, hinting at a mesenchymal stem cell-like phenotype [50]. However, another major population comprising 61.9 ± 3.6% showed expression of αSMA, which is a key SMC marker (Fig. 1c, left panel). The presence of significant amounts of nonspecific cell populations made it necessary to enrich the culture of endothelial cells by sorting. CD31 was chosen as a marker for enrichment to ensure a high enough quantity of mature EC.

**Fig. 2:**
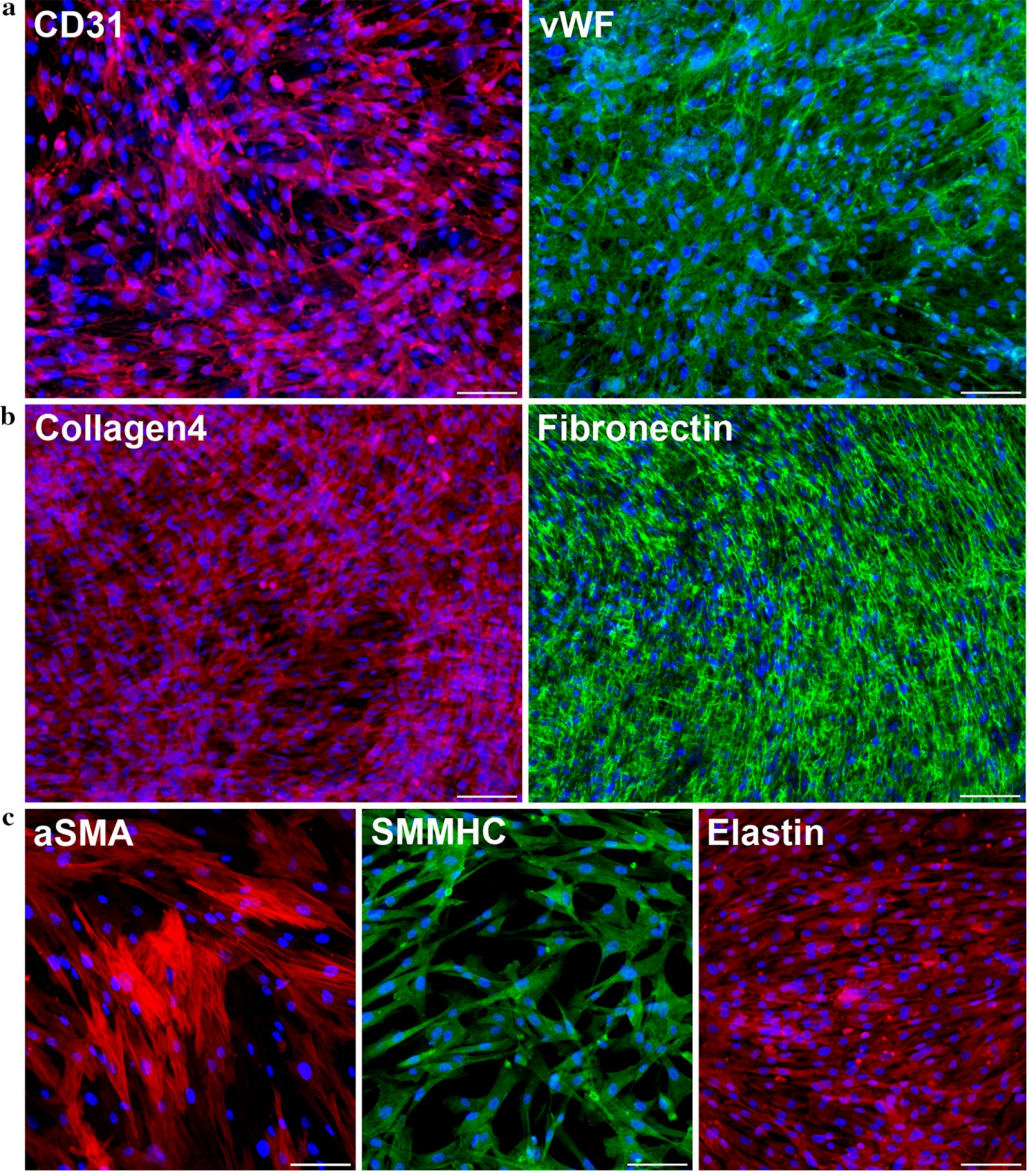
Properties of endothelial and smooth muscle cells after selection. **a** Endothelial cells at second passage after enrichment by MACS separation reveal CD31- and vWF-positive staining and produce extracellular matrix (collagen IV- and fibronectin-positive staining) (**b**). **c** Smooth muscle cells at the fifth passage reveal αSMA- and SMMCH-positive staining and produce extracellular matrix elastin. *Scale bar* 100 μm

After enrichment for EC by magnetic sorting, another round of FACS detected 72.6 ± 3.3% CD31-positive cells in the culture (Fig. 1c, left panel, Additional file 1: Table S1), which was maintained for at least 10 passages (data not shown). Furthermore, after sorting, the proportion of VEGFR2 (vascular endothelial growth factor receptor 2)-positive cells increased tenfold from 6.2 ± 1.6% in the unsorted population to 60.4 ± 2.5%, and mesenchymal marker CD90-positive cells dropped to a marginally trace level, as did α-SMA-positive cells. The results of immunofluorescent staining (Fig. 2a) also confirmed population enrichment of cells expressing mature EC-specific markers–CD31 and von Willebrand factor (vWF).

Correct endothelial cell monolayer formation also requires production of specific extracellular matrix components. Immunofluorescent staining showed that cells after enrichment of the EC population produced high levels of the matrix proteins fibronectin and collagen IV (Fig. 2b).

As for the primary culture that was grown in SmGM-2, even without sorting, we found that a majority of cells expressed an SMC marker (α-SMA), and this remained the case for more than 10 passages. Our FACS data indicated that at passage 5 96.1 ± 2.2% of cells cultured in SmGM-2 were α-SMA positive (Fig. 1c, right panel; Additional file 1: Table S1). It should be emphasized that, in FACS, a very low percent of endothelial marker-positive cells was detected under these culture conditions, and by passage 5 they completely disappeared. Furthermore, the quantity of CD90-positive cells decreased as well. However, as for CD90-positive cells, they composed 18.9 ± 2.7% of the population in SmGM-2 at passage 5, and immunostaining of cells maintained in SmGM-2 showed vivid production of smooth muscle myosin heavy chain (SMMHC) and elastin, a crucial component of vascular extracellular matrix component (Fig. 2c).

### Immunosorted cells from cardiac explants show ultrastructural and functional properties of mature EC

**Fig. 3:**
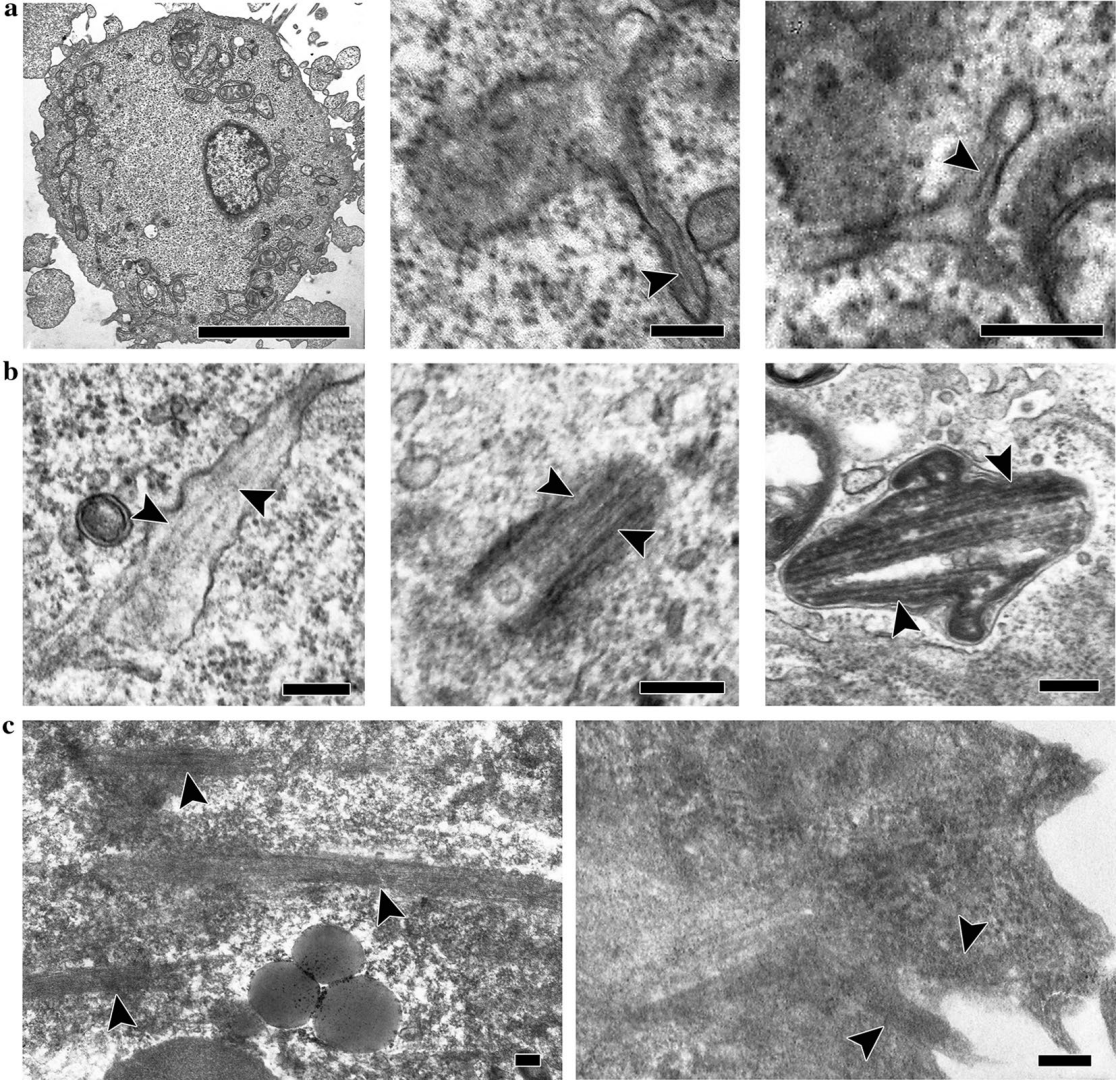
Dynamics of Weibel-Palade body (WPB) formation in the cytoplasm of endothelial cells, as analysed by transmission electron microscopy. **a** General view of endothelial cell ultrastructure; *scale bar* 5 µm (*left*). Early stage of WPB formation from Golgi apparatus in the cells on the 2nd day after sorting (*middle* and *right*); *scale bar* 100 nm. **b** Immature WPBs with small quantities of folded vWF structures (*left*) and mature WPBs containing significant amounts of vWF (*middle* and *right*) detected on the 4th day after sorting; *scale bar* 100 nm. **c** The secretion of vWF-folded structures from mature WPBs to the extracellular space on the 8th day after sorting; *scale bar* 100 nm. WPBs are indicated by the *arrowhead*

Using transmission electron microscopy, we demonstrated the presence of endothelial-specific microvesicles—Weibel-Palade bodies (WPBs)—in sorted CD31+ cells’ cytoplasm (Fig. 3). We managed to detect WPB formation (maturation) dynamics in endothelial cells in vitro. For that purpose, cells were analysed on the 2nd, 4th and 8th days after magnetic sorting. The early stages of WPB formation from the Golgi apparatus were detected on the 2nd day after EC sorting (Fig. 3a, middle and right). On the 4th day after sorting, we observed immature bodies with small numbers of folded vWF structures (Fig. 3b, left) as well as mature ones containing significant numbers of vWF (Fig. 3b, middle and right) in the cytoplasm of the cells. On the 8th day after sorting, we detected WPB membrane fusion with the cytoplasmic membrane and secretion of vWF-folded structures to the extracellular space (Fig. 3c).

Moreover, we have shown that sorted endothelial cells were able to metabolize acetylated low-density lipoprotein (acLDL), a characteristic ability of endothelial cells [51].

**Fig. 4:**
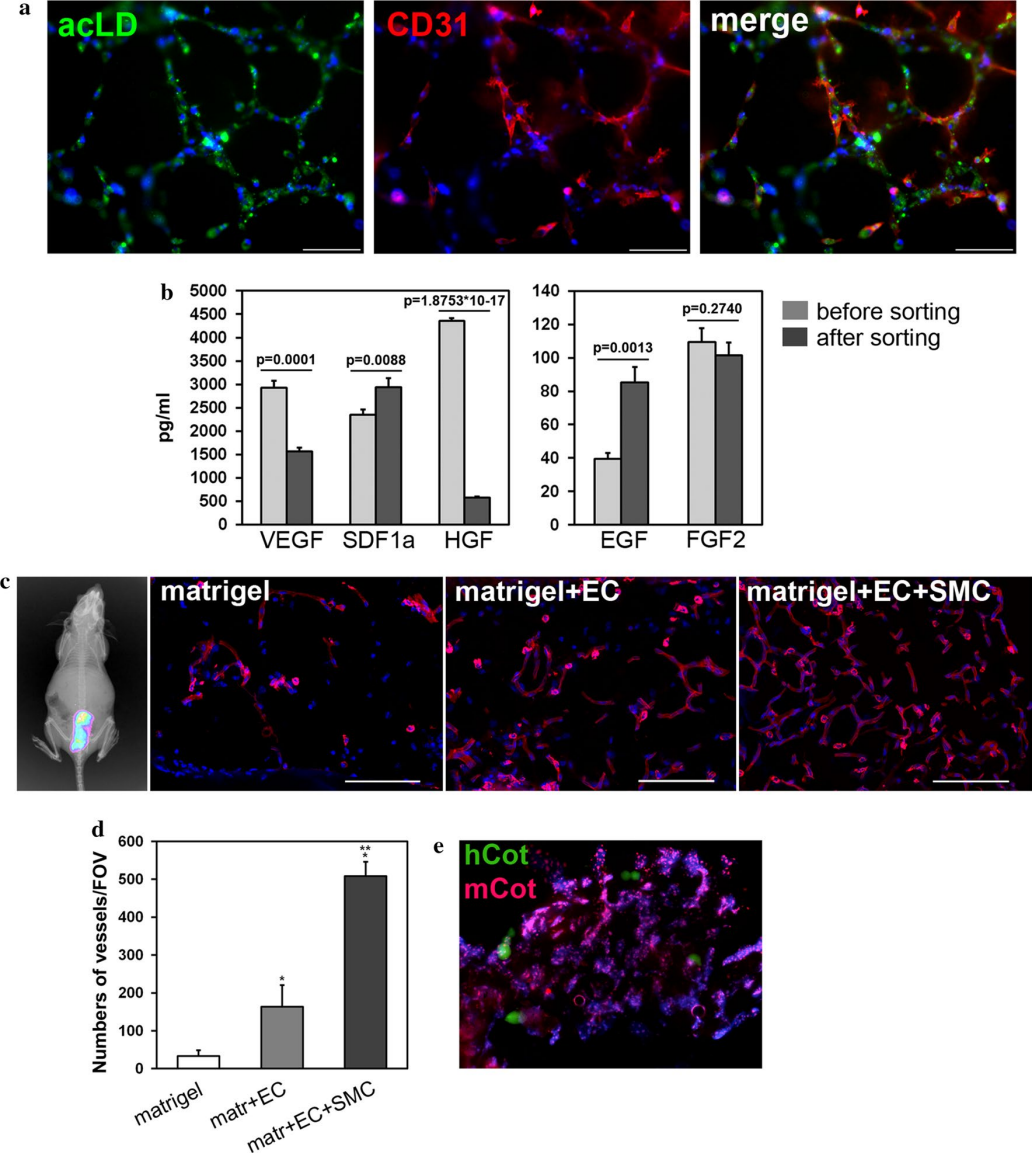
Functional properties of cardiac explant-derived cells. **a** Endothelial cells form capillary-like structures in Matrigel, uptake acLDL (*left*) and demonstrate a CD31-positive phenotype (*middle*). A merged image is represented in the picture on the* right*. *Scale bar* 100 μm. **b** Angiogenic cytokine profiles of conditioned medium obtained from cardiac explant-derived cells before and after MACS separation of CD31-positive cells. Wilcoxon test. **c** Evaluation of functional properties of CD31-positive endothelial cells in vivo. Visualization of injected mix (Matrigel + cells with the vital dye MitoTracker Deep Red FM) after 14 days with a Kodak In-Vivo Multispectral Imaging System device (*left*). Vasculature in cryosections of the Matrigel plug at day 14 after injection of Matrigel only, Matrigel + endothelial cells and Matrigel + endothelial + smooth muscle cells is detected by isolectin B4 Alexa 594 conjugate staining. *Scale bar* 100 μm. **d** A *diagram* representing the number of vessels positive for isolectin B4 Alexa 594 conjugate staining (*red colour*) as determined under a fluorescence microscope in 10 random fields of view. Wilcoxon test with Bonferroni correction. * vs Matrigel only, p = 0.0005; ** vs Matrigel + endothelial cells, p = 0.00003. **e** Fluorescent in situ hybridization of human and mouse DNA probes (hCot and mCot, respectively) to cryosection of Matrigel + EC + SMC. Human probes (*green*) are distributed evenly over the nuclei, whereas mouse probes (*red*) are detected in discrete dots due to the structure of α-satellite repeats in a mouse genome. Nuclei are counterstained with DAPI

The other test is based on the ability of endothelial cells to form tubule capillary-like structures in Matrigel. The in vitro tubule assay is routinely and extensively used as a functional test for endothelial cells. It is regarded as representative of in vivo capillary development [52, 53]. We have shown that sorted endothelial cells actively formed capillary-like structures in a 3D assay using Matrigel (Fig. 4a). The formed capillary-like structures were found to be CD31-positive after being fixed and stained by antibodies against the EC surface marker CD31.

We also assayed conditioned medium samples from unsorted culture and enriched EC populations for angiogenic cytokines. Evaluation of the profile of selected cytokines illustrates the functionality of sorted endothelial cells in their ability to induce paracrine effects [54–56]. ELISA results show a statistically significant decrease of VEGF and HGF content in conditioned medium obtained from the CD31-enriched population (Fig. 4b; Additional file 1: Table S2). Opposite to this, SDF-1a and EGF content was increased after EC enrichment compared to unsorted culture (Fig. 4b; Additional file 1: Table S2). Regarding FGF-2, a trend towards decrease of this cytokine was observed in sorted EC conditioned medium, but it was statistically insignificant (109.53 ± 8.19 pg/ml in unsorted vs 101.28 ± 7.75 pg/ml in sorted endothelial cells; p = 0.274).

### In vivo test for angiogenic potential of vascular cell cultures from cardiac explants

The vascularization of a subcutaneously injected Matrigel mixed with tested cells was used to examine their angiogenic potential in vivo. The Matrigel plug assay is currently a conventional test confirming the functionality of endothelial and smooth muscle cells in vivo [57, 58]. The formation of networks of EC tubes is a process that appears to be specific to these cells and mimics the formation of capillary networks in vivo [59].

An enriched culture of CD31-positive EC was tagged with a vital dye (MitoTracker Deep Red) to visualize injected cells. Viable cells were visualized in the area of injection after 14 days with a Kodak In-Vivo Multispectral Imaging System device (Fig. 4c), and vasculature was visualized in frozen sections of Matrigel plugs stained with isolectin B4. Fluorescent isolectin-positive capillary structures were present on day 14 after implantation (Fig. 4c) quantitative analysis revealed that vessel counts were significantly higher in specimens where Matrigel was mixed with CD31-enriched cardiac explant cells compared to the control of Matrigel mixed with the plain vehicle, PBS (Fig. 4d; 164 ± 57 vs 33 ± 15 structures/FOV; p = 0.0005). In histology preparations from Matrigel plugs that contained a mix of EC and SMC, this parameter reached a maximum value of 508 ± 38 structures/FOV, which was higher than in either EC or acellular transplants (Fig. 4d).

To evaluate human cell retention, we used FISH in prepared Matrigel + EC + SMC sections and detected transplanted human and host murine cell nuclei (Fig. 4e). Note that enhancement of angiogenesis after injection of cells is considered a paracrine effect of injected cells [9, 15, 25]. In studies, the angiogenic potential of injected cells is tested by this method. The human injected cells remaining at 14 or more days after the injection have been visualized by either HNA antibodies or human CD31-specific antibodies. In our experiment, we have shown the presence of the remaining injected human cells by FISH, because this method is reliable, detects species-specific DNA repeats and does not require matching antibodies to prevent antibodies made in mice from cross-precipitating to mouse cells on the slide.

### Endothelial and smooth muscle cell application on polycaprolactone and chitosan membranes: Proliferation and viability investigation

Based on previously established protocols for manufacture of chitosan/PCL membranes [8, 43, 45, 60–65], we prepared several membranes with different chitosan/PCL ratios (see Materials and Methods for details) (Additional file 2: Figure S1a). In the process of mixture making, the source components are solved in acetic acid, and the final polymer membranes are neutralized by alkaline solution. Recent data suggest that mammalian cells proliferate more actively on chitosan surfaces neutralized by alkali mixed with ethanol rather than after neutralization by alkali alone [44]. The neutralization method affects the nanostructure of the resulting polymer’s fibres, which, in turn, influences the membrane surface topography and the populated cells’ adhesive capacity [66]. Thus, chitosan membrane neutralization by NaOH solution in 80% ethanol gives advantages in mouse fibroblast adhesion and proliferation compared to neutralization by aqueous NaOH solution. However, that fact was not explored for PCL and chitosan mixtures. It is also known that the ratio of PCL-chitosan mixture components affects cell proliferation dynamics [67, 68].

**Fig. 5:**
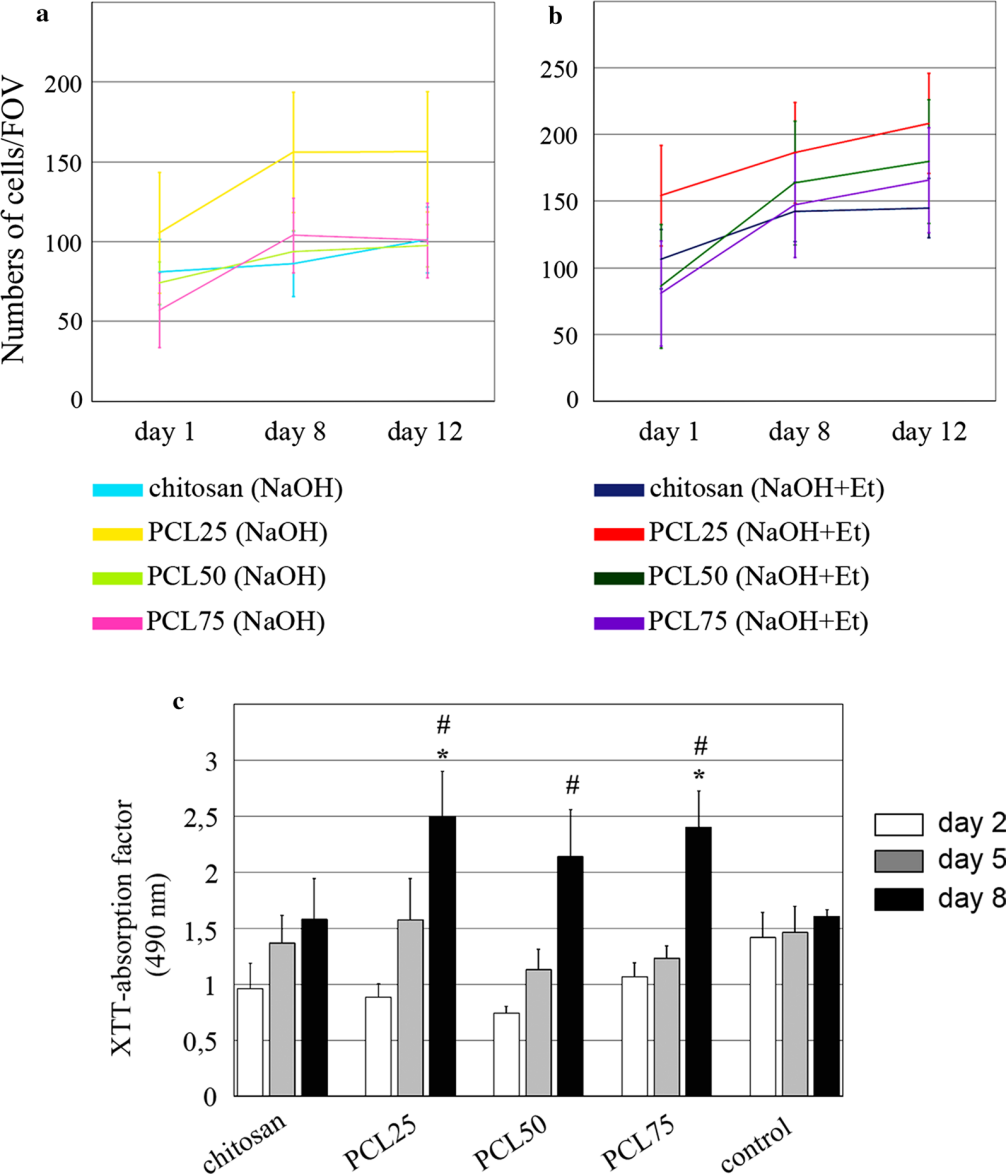
Dynamics of proliferation and viability of CD31-positive endothelial cells cultured on chitosan, PCL25, PCL50 and PCL75. **a** Numbers of cells in five random fields of view (FOVs) after 1, 8 and 12 days of cultivation on membranes neutralized by alkali (NaOH). **b** Numbers of cells in five random fields of view (FOVs) after 1, 8 and 12 days of cultivation on membranes neutralized by alkali mixed with ethanol (NaOH + Et). **c** XTT assay of CD31-positive endothelial cells cultured on membranes and on untreated plastic dishes (control) for 2, 5 and 8 days. Wilcoxon test. * vs chitosan day 8, p < 0.01;^ #^ vs control day 8 p < 0.01

Our comparative data using both types of neutralization procedure shows that after 12 days of culture on PCL25 chitosan/PCL membranes treated with a mixture of NaOH/ethanol, CD31+ -sorted EC showed significantly better proliferation dynamics compared to NaOH neutralization (p < 0.01, Figure S1b in Additional file 2). A similar trend was observed for PCL50 and PCL75, but this difference did not reach statistical significance at any timepoint (Fig. 5b; Additional file 2: Figure S1b).

As for differences between different chitosan/PCL membranes treated with NaOH/ethanol, PCL25 showed optimal performance compared to PCL50 and PCL75 (p < 0.01). In the case of alkaline neutralization this was not reproducible, and we observed comparable proliferation of EC on PCL25/50/75 with a non-significant tendency towards a PCL25 advantage over other compositions (Additional file 2: Figure S1b).

The improvement of cell proliferation on chitosan/PCL membranes compared to chitosan alone (taken as 100%) was confirmed by XTT analysis of cells seeded on NaOH/ethanol-treated membranes for 2, 5 or 8 days (Fig. 5c). By the 8th day of observation, the number of viable cells on PCL25 and PCL75 was significantly higher than on chitosan (Fig. 5c), and on all tested membranes cell counts were significantly higher compared to uncoated culture dishes. Thus, for subsequent experiments using human cells seeded on membranes, we used NaOH/ethanol-treated PCL25 membranes.

### In vitro analysis of EC functional properties in PCL25 chitosan/PCL membrane

**Fig. 6:**
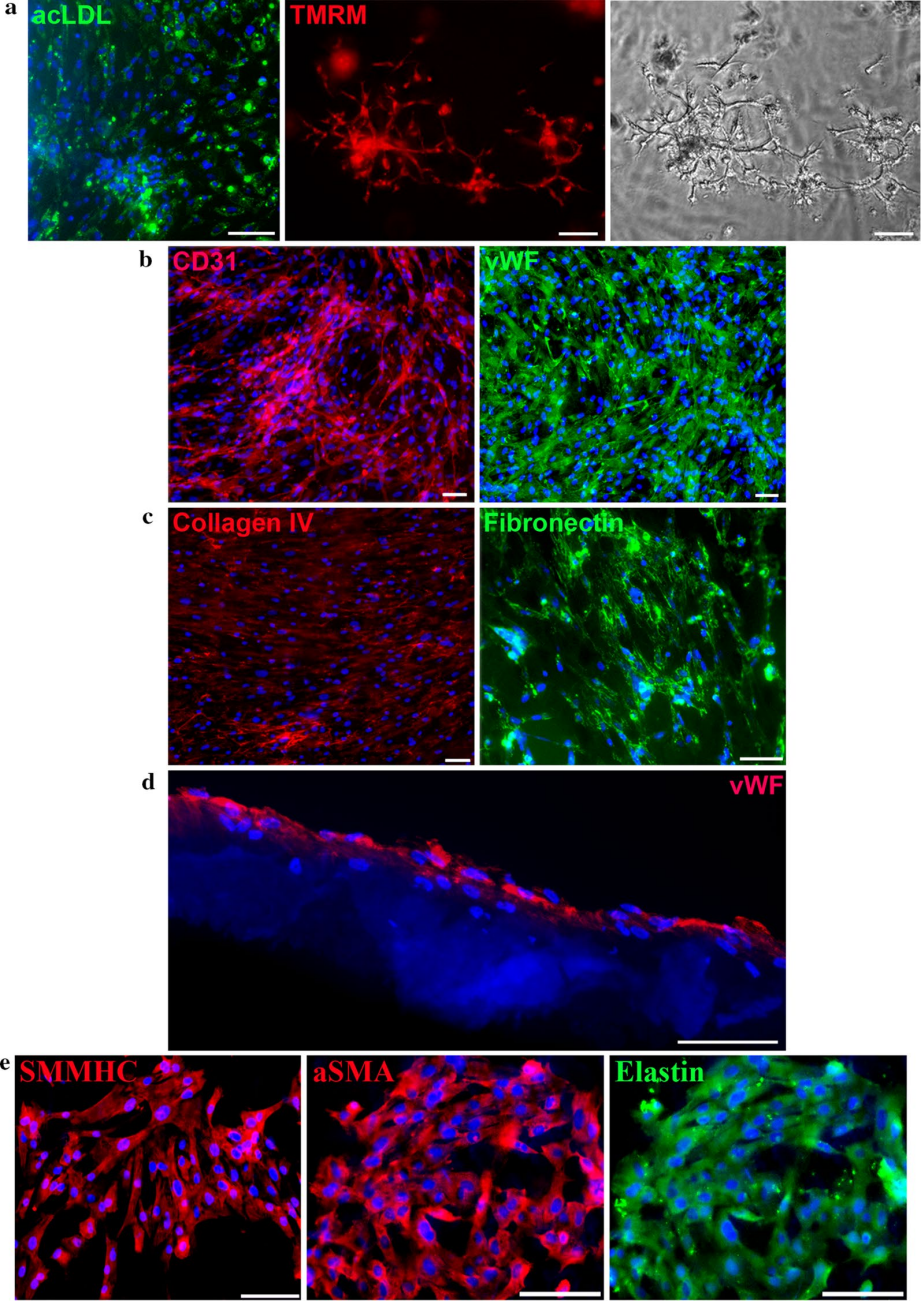
Properties of cardiac explant-derived endothelial and smooth muscle cells cultured on PCL25 membranes (except Fig. 6d) neutralized by alkali mixed with ethanol. **a** Endothelial cells cultured on PCL25 uptake acLDL (*left*), form capillary-like structures in Matrigel (*middle* cells stained with vital dye TMRM; *right* phase contrast image). *Scale bar* 100 μm. **b** Endothelial cells cultured on PCL25 retain specific endothelial markers: CD31 (*left*), vWF (*right*). *Scale bar* 50 μm. **c** Endothelial cells cultured on PCL25 retain the ability to produce extracellular matrix (collagen IV- and fibronectin-positive staining). *Scale bar* 50 μm. **d** Cryosection over cell-seeded PCL75 membrane. Monolayer of endothelial cells has vWF-positive staining. *Scale bar* 20 μm. **e** Smooth muscle cells cultured on PCL25 retain specific smooth muscle markers: SMMCH (*left*), αSMA (*middle*), elastin (*right*)

Maximum biocompatibility of tissue-engineered constructs and imitation of physiological features depend on the seeded cells’ functional properties and the stability of the cells’ features after they are seeded onto the matrix. Since cells demonstrated optimal proliferation and viability on PCL25 surfaces, our subsequent estimations of cell functional properties were made on this type of membrane. Endothelial cells populated on PCL25 retained the ability to metabolize acLDL and to form capillary-like structures in the depth of 3D Matrigel culture (Fig. 6a). The cells composing studied tissue-engineered constructs retain the specific endothelial markers CD31 and von Willebrand factor (Fig. 6b), and they also yield the extracellular matrix components fibronectin and collagen IV (Fig. 6c). In addition, on sections of cell-seeded tissue-engineered constructs, the formed endothelial cell monolayer stained vividly for vWF (Fig. 6d). We have also shown that SMC keep their specific markers, αSMA and SMMHC, and yield elastin—a component of the extracellular matrix—when populated on a surface made of the polycaprolactone and chitosan mixture PCL25 (Fig. 6e).

## Discussion

Methods for isolation of cardiac EC and SMC have been described for many animal models, namely, mouse, rat, guinea pig, and rabbit [69–78]. All of them are based on either a perfusion technique or enzymatic hydrolysis of myocardial samples, excised from the isolated heart from sacrificed animals. Isolation of human cardiac endothelial cells was described by Gräfe et al. and McDouall et al. [37, 38]. As a source of endothelial cells, they used explanted human hearts of recipients who underwent cardiac transplantation. The first method requires a specialized perfusion system; the second was based on fermentative hydrolysis of chunks of ventricle from an explanted human heart followed by sorting with an anti-HLADR antibody or Ulex lectin-coated magnetic beads. The authors emphasize the importance of studying cardiac endothelial cells to investigate the pathophysiology/pharmacology of cardiovascular disease [37].

The atrial appendage as a post-operative by-product is widely used for producing c-kit-positive cardiac stem cells (CSC), whose regenerative potential has been the focus of extensive research, including clinical trials (SCIPIO, CADUCEUS—see clinicaltrials.gov) [79–82]. We propose to use this source as well as post-operative myocardial material to isolate human EC and SMC, which can be a source of patient-specific therapeutic cell populations in patients who face the prospect of surgical reoperation.

We introduced a technique for functional endothelial and smooth muscle cell extraction from post-operative myocardial specimens taken from the right atrial appendage and right ventricle. The proposed method includes the following stages: enzymatic tissue hydrolysis, culture of isolated cells on specific media on the surface of human collagen IV, enrichment by means of magnetic sorting for an endothelial population with antibodies against the mature endotheliocyte marker CD31, and further cultivation on a surface processed with collagen IV in specific culture media.

After enrichment by means of magnetic sorting, the content of CD31-positive cells increases more than fourfold, and the content of VEGFR2-positive cells increases almost tenfold in medium for endotheliocyte growth. Along with that, the content of CD90-positive cells non-specific for an endothelial population decreases up to 2.1 ± 2.8%, and αSMA-positive cells are lost. It is worth noting that in the process of primary culture in medium without added growth factors, endothelial markers are completely lost after 2–3 passages, and the fraction of CD90-positive cells increases up to 80% (data not presented). It is confirmed by a number of further tests that the population enriched for the endothelial marker CD31 represents functional endotheliocytes.

One important aspect of the functional characterization of an endothelial population is an ultrastructural study for the presence of typical granules. WPBs are distinctive cigar-shaped granules specific to functional endothelial cells [83]. WPBs contain molecules of vWF protein, P-selectin, and the other functional molecules secreted by endothelial cells in response to shifting microenvironment conditions [84, 85]. Thus, ultrastructural study of these cells reveals the presence and formation dynamics of WPBs (Fig. 3). In mature WPBs, folded structures of vWF—one of the basic components contained in the granules—are detected. The ability of derived endothelial cells to produce the extracellular matrix components fibronectin and collagen IV, to form capillary-like structures in Matrigel and to metabolize the acetylated form of low density lipoprotein is shown in the present study.

In terms of the investigation of cells’ functional properties by means of ELISA, cytokine quantities were estimated in conditioned medium obtained from primary unsorted culture, grown in EGM-2, as well as enriched endotheliocyte culture. The discovered decrease of VEGF165 in conditioned medium obtained from sorted cells compared to unsorted can be associated with two factors. First, cytokines are expressed not only by endothelial cells but by a number of other cell types (macrophages, activated T-lymphocytes and others) [86, 87], which can be present in primary unsorted population. Direct enrichment of endothelial cells leads to the loss of a significant portion of other VEGF-secreting cells. For example, the fraction of CD90-positive cells decreases more than 20-fold. Second, the decrease of VEGF165 content in conditioned medium from sorted endotheliocytes can be associated with the almost tenfold increase of cells containing the VEGF receptor VEGFR2. FACS data indicate that the VEGFR2-positive portion elevates from 6.2 ± 1.6% in an unsorted population to 60.4 ± 2.5% in a sorted one. An analogous tendency to decrease in conditioned medium from enriched endotheliocytes is observed for HGF. Additionally, an insignificant drop of FGF-2 level is detected. The opposite tendency is revealed for SDF-1α and EGF. Their content in conditioned medium from sorted endothelial cells is reliably higher than in medium obtained from an unsorted cell population. A higher content of the given chemokines indicates the endothelial cells’ paracrine potential.

Functional characteristics in vitro were confirmed by functional testing in vivo on an abdominal Matrigel implant model in immunodeficient SCID mice. It has been shown by Japanese authors [9, 15], that combined transplantation of human EC and mural cells (MC, both smooth muscle cells and pericytes) could markedly increase vascular regeneration compared to single-fraction transplantation of EC or MC. However, a transplantation of mural cells leads to less pronounced revascularization compared to endothelial cells. Therefore, to test in vivo the functional properties of the derived endothelial and smooth muscle cells, we had three experimental groups of SCID-mice: injection of endothelial cells, injection of both smooth muscle endothelial cells, and PBS as a control. Injection of endothelial cells leads to fivefold multiplication of vascular structures detected on Matrigel implant cryosections in comparison with control PBS injection. After cooperative injection of endothelial and smooth muscle cells derived from human myocardium, the quantity of vascular structures increases 15-fold compared to the control (Fig. 4d).

We have shown by fluorescent in situ hybridization that cryosections of Matrigel implants contain not only human cells but also mouse cells, the latter to a larger extent (Fig. 4e). This is affirmed by paracrine factor action, which facilitates the development of vascular structures composed of mouse cells in response to the introduction of human endothelial cells.

Thus, we proposed a new source for functional endothelial and smooth muscle cells isolation—post-operational material from the human right atrial appendage and right ventricle myocardium. These cells can be used for the development of cell-seeded tissue-engineered constructs for the purposes of vascular surgery. As an example of the application of both types of cells, we seeded them on a biodegradable scaffold—a polycaprolactone/chitosan (PCL/CH) blended membrane. We characterized the morphological and functional properties of those cells and evaluated their biological behaviour after seeding to chitosan/PCL membranes.

Blood vessel substitutes from biological and synthetic materials have been extensively developed in recent years [66, 88]. Vascular grafts made of polytetrafluoroethylene (PTFE), polyethylene terephthalate (PET), and polyurethanes have been widely tested, and in spite of synthetic materials’ positive features (accurate sizing, permeability, controlled structure and mechanical properties), their application brought unsatisfactory long-term results [88]. The latter is especially an issue when synthetic prostheses of small diameter are used. The complications include stenosis, thrombosis, neointimal hyperplasia, lack of vascularization and inflammation induced by polymer degradation products resulting in loss of structural integrity [89, 90]. This problem drew researchers’ attention to biodegradable materials, among which chitosan and polycaprolactone (PCL) found wide medical application [60]. Using a mixture of natural (chitosan) and synthetic (PCL) polymers made it possible to combine positive features of both materials [91]. Furthermore, this approach increases feasibility and reduces expense compared to standard PCL-monomer synthesis followed by polymerization. In a number of works, both chitosan and PCL separately were found to be inferior to their combination, with optimal proportions of these components established and tested in membranes that were successfully seeded with mouse fibroblasts [45], bovine corneal endothelial cells [43] or human dermal microvascular EC [44]. The possibility of using material consisting of a mixture of polycaprolactone and chitosan, seeded with autologous peripheral blood mononuclear cells, to create a small diameter vessel has been demonstrated in vivo in a canine model [8]. However, even combined chitosan/PCL material has been shown to have much room for improvement, and as a mixture of 2 polymers gave better functional properties, seeding these with several ex vivo cultured cell types can provide more reliable reproduction of the properties of blood vessels.

The proliferation dynamics and retention of specific functional properties of endothelial and smooth muscle cells of human cardiac explants, contained in tissue-engineered constructs made on a base of polycaprolactone and chitosan, were analysed in vitro. We have shown that a mixture of polycaprolactone and chitosan is advantageous for endothelial cell proliferation in comparison with 100% chitosan. The proliferation indices of the endothelial cells tends to be higher upon alkaline-ethanol solution neutralization compared to alkaline aqueous solution (Fig. 5b). Cells proliferate on PCL25 surfaces more effectively than on PCL50 or PCL75 surfaces. Therefore, it is preferable to use membranes with a 25:75 ratio of PCL and chitosan, neutralized with a mixture of alkaline and ethanol, when creating tissue-engineered constructs with endothelial cells from human cardiac explants.

An important aspect to confirm the functionality of the cells growing on the scaffold is the retention of a typical pattern of cell antigens. It has been shown that, depending on the composition of the scaffold, the pattern of markers may change, even to the point that some of them are lost entirely. For example, the integral membrane protein Na–K ATPase in bovine corneal endothelium, growing on substrates of mixtures with different ratios of PCL/CH, was only weakly detected on PCL5 and PCL10, while levels appeared to be normal on PCL15 and PCL25 [43]. It is reported that, when cells are grown on 100% chitosan, this marker is completely lost in the cells. It has been shown that, if human dermal microvascular endothelial cells (HDMEC) are grown on the surface of the metal alloy Co28Cr6Mo, endothelial cells acquire an aberrant pattern of intracellular distribution of CD31, VE-cadherin, ZO-1 and F-actin [92]. In experiments conducted in vivo, implants of CoCrMo-alloy disrupted the integrity of the microvascular endothelium in the surrounding tissues and caused extensive oedema [93], whereas the cultivation of HDMEC on the surface of a titanium alloy does not lead to changes in the pattern of intracellular distribution of these markers [92].

We have shown in vitro retention of specific markers contained in endothelial and smooth muscle cells in tissue-engineered constructs on a PCL and chitosan mixture. For endothelial cells grown on membranes made of given polymer mixtures, retention of CD31 and vWF was shown, along with the capacity to metabolize low-density lipoproteins, to yield the extracellular matrix components collagen IV and fibronectin, and to form capillary-like structures in Matrigel poured onto cells sitting on the membrane. For smooth muscle cells, αSMA and SMMHC retention was shown. Retention of cell functional properties in the composition of tissue-engineered constructs in vitro gives grounds for the assumption that populating a mixed polymer membrane would facilitate adequate integration to surrounding tissues and minimize negative remote results for vascular transplant. Thus, in the year 2013, a group of investigators demonstrated the possibility of using material composed of a mixture of PCL and chitosan for cell-seeded vessels of small diameter (less than 6 mm) [8]. The nano-fibres utilized in scaffold formation were obtained by electro-spinning of given polymer mixture, and then they were populated by autologous mononuclear cells from canine peripheral blood and implanted in the carotid arteries of 6 dogs. Histological and immunohistochemical analysis of the tissue-engineered constructs, obtained after 3 months, showed the regeneration of endothelium and the presence of collagen and elastin [8]. In work on bovine corneal endothelium growing on membranes prepared from PCL-chitosan mixture by means of evaporation in a convection oven, the authors demonstrated the high proliferative capacities and preservation of morphophysiological and functional properties of endothelial cells in the constructs [43].

## Conclusions

In the present study, we established a method for isolation, culture and enrichment of endothelial and smooth muscle cells from post-operative cardiac tissues from the right atrial appendage and right ventricle. This method allows one to obtain a significant number of cells that reveal functional properties in vitro and demonstrate angiogenic potential in vivo. As an example of how cells isolated by the proposed method may be used for tissue engineering, we seeded the cells on a biodegradable scaffold—a membrane consisting of a blend of polycaprolactone and chitosan. Isolated endothelial and smooth muscle cells may be a promising source of patient-specific cells for regenerative medicine.

## Additional files

**Additional file 1.** Flow cytometric analysis of CD31, VEGFR2, CD90 and αSMA expression (**Table S1**) and Quantification of angiogenic cytokines (**Table S2**) in cardiac explant-derived cells. **Table S1**. Flow cytometric analysis of CD31, VEGFR2, CD90 and αSMA expression in cardiac explant-derived cells. Comparison of cells cultivated in endothelial growth medium before and after MACS-separation. Comparison of cells cultivated in smooth muscle cells growth medium at the second and fifth passages. **Table S2.** Quantification of angiogenic cytokines in cardiac explant-derived cells cultivated in endothelial growth medium before and after MACS-separation.

**Additional file 2: Figure S1.** Membranes with different chitosan/PCL ratios and the dynamics of endothelial cell proliferation on them. **a.** A general view of Petri dishes with an untreated surface and surfaces with transparent membranes made of chitosan, PCL25, PCL50, PCL75. **b.** A summary diagram showing the numbers of endothelial cells in five random fields of view (FOVs) after 1, 8 and 12 days of cultivation on membranes of chitosan, PCL25, PCL50, and PCL75 neutralized by alkali (NaOH) or alkali mixed with ethanol (NaOH + Et).

## Abbreviations

acLDL: acetylated low density lipoprotein
αSMA: α-smooth muscle actin
BSA: bovine serum albumin
DOP-PCR: degenerate oligonucleotide primed PCR
EC: endothelial cells
EGM-2: Endothelial Cell Growth Medium-2 (Lonza)
FACS: fluorescence-activated cell sorting
FISH: fluorescent in situ hybridization
FOV: fields of view
HDMEC: human dermal microvascular endothelial cells
MACS: magnetic-activated cell sorting
PBS: phosphate-buffered saline
PCL: polycaprolactone
PFA: paraformaldehyde
PTFE: polytetrafluoroethylene
SMC: smooth muscle cells
SmGM-2: Smooth Muscle Growth Medium-2 (Lonza)
SMMHC: smooth muscle myosin heavy chain
SSC: saline-sodium citrate
TMRM: tetramethylrhodamine, methyl ester
WPBs: Weibel-Palade bodies
XTT: (2,3-bis-(2-methoxy-4-nitro-5-sulfophenyl)-2H-tetrazolium-5-carboxanilide)
