# Supplementary material for: Endothelial and smooth muscle cells derived from human cardiac explants demonstrate angiogenic potential and suitable for design of cell-containing vascular grafts
Source: J Transl Med. 2017 Mar 3;15:54. doi: 10.1186/s12967-017-1156-1 (PMC5336693; doi:10.1186/s12967-017-1156-1)
Supplement: Supplementary file 1 — Additional file 1. Flow cytometric analysis of CD31, VEGFR2, CD90 and αSMA expression (Table S1) and Quantification of angiogenic cytokines (Table S2) in cardiac explant-derived cells. Table S1. Flow cytometric analysis of CD31, VEGFR2, CD90 and αSMA expression in cardiac explant-derived cells. Comparison of cells cultivated in endothelial growth medium before and after MACS-separation. Comparison of cells cultivated in smooth muscle cells growth medium at the second and fifth passages. Table S2. Quantification of angiogenic cytokines in cardiac explant-derived cells cultivated in endothelial growth medium before and after MACS-separation. [file 12967_2017_1156_MOESM1_ESM.docx]

Table S1. Flow cytometric analysis of CD31, VEGFR2, CD90 and αSMA expression in cardiac explant-derived cells. Comparison of cells cultivated in endothelial growth medium before and after MACS-separation. Comparison of cells cultivated in smooth muscle cells growth medium at the second and fifth passages.

| marker | % of positive cells  in EGM | | % of positive cells  in SmGM | |
| --- | --- | --- | --- | --- |
|  | before sorting | after sorting | passage 2 | passage 5 |
| CD31 | 16,1±2,1 | 72,6±3,3 | 8,6±1,5 | 0,0 |
| VEGFR2 | 6,2±1,6 | 60,4±2,5 | 2,5±1,3 | 0,0 |
| CD90 | 44,7±2,5 | 2,1±2,8 | 63±2,6 | 18,9±2,7 |
| αSMA | 61,9±3,6 | 0,0 | 87,5±2,2 | 96,1±2,2 |

Table S2. Quantification of angiogenic cytokines in cardiac explant-derived cells cultivated in endothelial growth medium before and after MACS-separation.

| marker | values, ng/ml±SD | |
| --- | --- | --- |
|  | before sorting | after sorting |
| VEGF165 | 2931,00±149,43 | 1567±74,69 |
| SDF-1a | 2345,00±115,33 | 2944±183,65 |
| HGF | 4354,00±65,76 | 580±15,05 |
| EGF | 39,53±3,49 | 85,34±9,21 |
| FGF-2 | 109,53±8,19 | 101,28±7,75 |
