# Supplementary figures and images for: Endothelial and smooth muscle cells derived from human cardiac explants demonstrate angiogenic potential and suitable for design of cell-containing vascular grafts
Source: J Transl Med. 2017 Mar 3;15:54. doi: 10.1186/s12967-017-1156-1 (PMC5336693; doi:10.1186/s12967-017-1156-1)

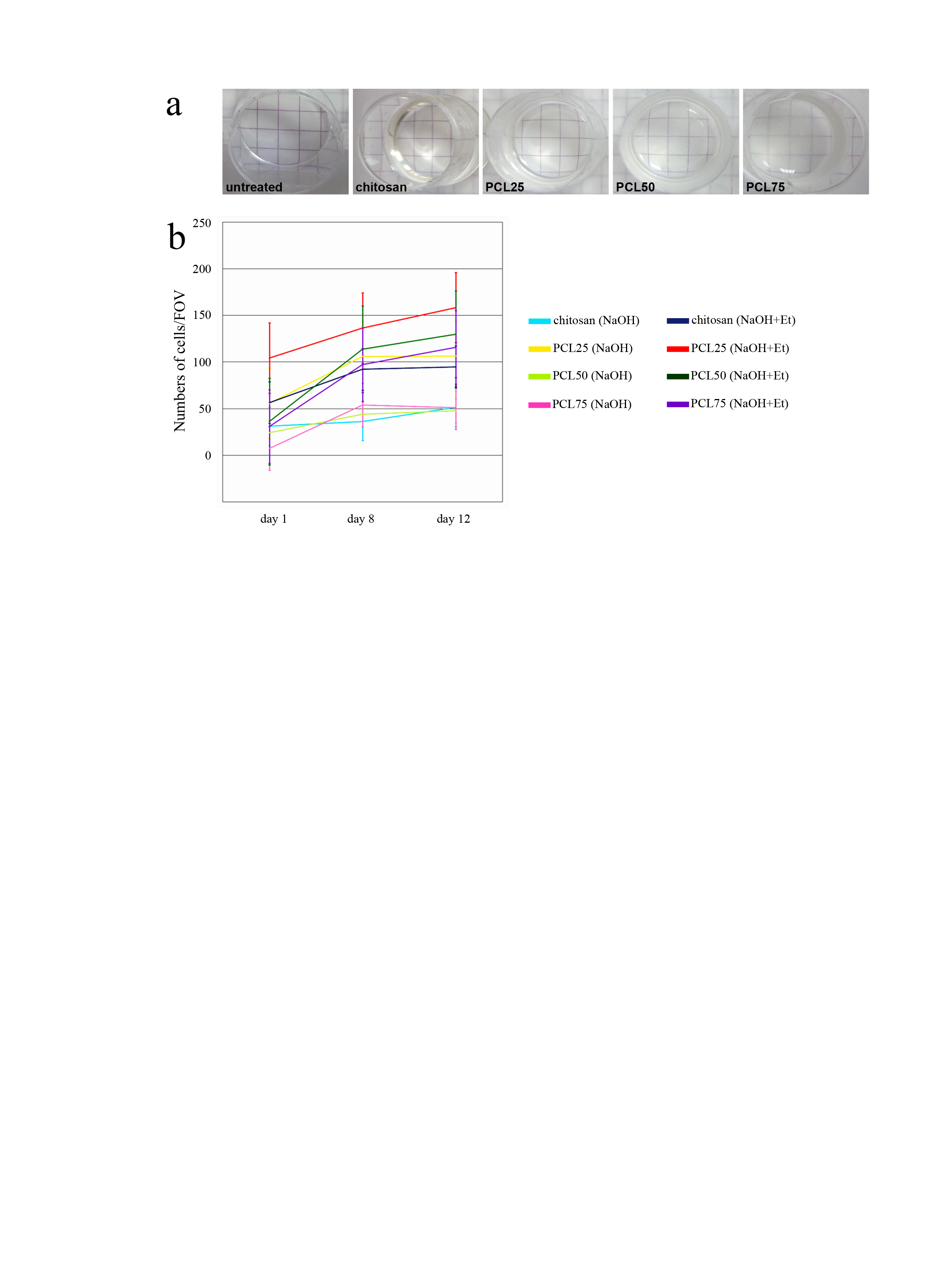

Supplement: Supplementary file 2 — Additional file 2: Figure S1. Membranes with different chitosan/PCL ratios and the dynamics of endothelial cell proliferation on them. a. A general view of Petri dishes with an untreated surface and surfaces with transparent membranes made of chitosan, PCL25, PCL50, PCL75. b. A summary diagram showing the numbers of endothelial cells in five random fields of view (FOVs) after 1, 8 and 12 days of cultivation on membranes of chitosan, PCL25, PCL50, and PCL75 neutralized by alkali (NaOH) or alkali mixed with ethanol (NaOH + Et). [file 12967_2017_1156_MOESM2_ESM.tif]
